# Sensitive Competitive Electrochemical Immunosensor for Hg (II) Based on Molybdenum Disulfide/Reduced Graphene Oxide/Gold Nanocomposites

**DOI:** 10.3390/s25030623

**Published:** 2025-01-22

**Authors:** Yuzhen Wang, Ningna Shi, Xiaoyue Kang, Qiliang Pan, Maozhong Tian, Yanfeng Wang, Yunfeng Bai

**Affiliations:** 1 College of Chemistry and Chemical Engineering, Shanxi Provincial Key Laboratory of Chemical Biosensing, Shanxi Datong University, Datong 037009, China; 15993089987@163.com (N.S.); 17803466537@163.com (X.K.); tmzhong2002@163.com (M.T.); sxwenfei@126.com (Y.W.); 2 Engineering Research Center of Coal-Based Ecological Carbon Sequestration Technology of the Ministry of Education, Shanxi Datong University, Datong 037009, China; pql0204@163.com

**Keywords:** electrochemical immunosensor, Hg (II), differential pulse voltammetry (DPV), molybdenum disulfide/reduced graphene oxide/gold (MoS_2_/rGO/Au) nanocomposites

## Abstract

A sensitive and specific competitive electrochemical immunosensor for the detection of Hg (II) using a modified electrode based on molybdenum disulfide/reduced graphene oxide/gold (MoS_2_/rGO/Au) nanocomposites was developed in this study. The nanocomposites were characterized and assembled with an antibody against Hg (II) for the immunosensor, demonstrating good electrical activity, high affinity and high specificity. Free Hg (II) in a solution can be measured by the competitive reaction of the Hg element in the sample and the antigen with the antibody fixed on the electrode. A differential pulse voltammetry (DPV) method was used, and the competitive current changed in accordance with the concentration of Hg (II). Under optimal conditions, the sensor showed a linear relationship from 0.1 to 600 ng/mL, and the limit of detection (LOD) was 63 pg/mL. The proposed immunosensor showed an acceptable recovery from 98.4% to 100.3% in spiked samples. Satisfactory stability and reproducibility were obtained. Competitive species, including Zn (II), Mg (II), Al (III), Cu (II), Pb (II), Ba (II), Cd (II), Ag (I), MNA, CH_3_Hg (I) and CH_3_Hg-MNA, were selected and applied according to the procedure of the assay, and their significantly different response compared to Hg (II) indicated that the assay displayed not only high sensitivity but also high selectivity. This immunosensor offers a useful model for the detection of Hg (II).

## 1. Introduction

As is well known, mercury is toxic and stable, and it is one of the most toxic elements among all the heavy metals. When released into the environment, it can damage human health [1]. Therefore, it is necessary to develop mercury detectors for both environmental and biological samples [2]. In the past few years, various methodologies for detecting mercury have been reported, such as cold-vapor atomic absorption spectrometry (CV-AAS) [3], cold-vapor atomic fluorescence spectrometry (CV-AFS) [4], inductively coupled plasma mass spectrometry (ICP-MS) [5], enzyme-linked immunosorbent assay (ELISA) [6] and electrochemical methods [7], etc. Recently, several sensors based on antibodies [8,9], DNA [10,11], urease [12], carbon nanodots [13] and covalent organic frameworks (COFs) [14] for detecting metal ions have also been reported.

As used in numerous methods, electrochemical biosensors are a method based on biological components, such as antigens/antibodies, enzymes, aptamers, proteins, whole cells, etc. Because of the strong specificity of biological components, these electrochemical biosensors have high selectivity. With the advantages of delivering convenient, rapid, sensitive, and simple operation, electrochemical biosensors have been widely applied to the detection of heavy metal ions [15,16,17,18,19,20,21]. The modification of the electrodes is particularly crucial for these sensors [22]. In the last decade, because of its attractive properties, such as a large surface area, high conductivity and excellent stability, reduced graphene oxide (rGO) has been extensively applied in electrochemical sensors [23,24]. On the other hand, graphene-based materials have been also widely used in electrochemistry due to their enhanced electrochemical characteristics, which could improve the activity of the electrode. Recently, the integration of molybdenum disulfide (MoS_2_) with graphene by different methods has been reported and applied in electrochemical sensors [25,26,27,28,29], exhibiting excellent electron conductivity. Here, rGO can not only be used as the carrier of molybdenum disulfide and gold nanoparticles, but it also has excellent electrochemical properties.

In our previous studies, we successfully prepared an antigen (methylmercury–6-mercaptonicotinic acid–bovine serum albumin, CH_3_Hg-MNA-BSA) and used the hybridoma technique to produce monoclonal antibodies against Hg (II), which were then characterized by an indirect competitive enzyme-linked immunosorbent assay (ELISA) [6]. Herein, we propose a sensitive and facile competitive electrochemical immunosensor for detecting Hg (II) based on MoS_2_/rGO/Au nanocomposites and a monoclonal antibody against Hg (II). MoS_2_ and MoS_2_/rGO were synthesized via a hydrothermal method and combined with Au nanoparticles, which showed excellent electrochemical properties due to their synergistic effects. The structures of the nanocomposites were analyzed by scanning electron microscopy (SEM), X-ray photoelectron spectroscopy (XPS) and X-ray diffraction (XRD). When the electrode was modified with the MoS_2_/rGO/Au nanocomposites, an immunoreaction occurred on the surface. The detection mechanism is based on the competition of the Hg element in the sample and the antigen with the antibody fixed on the electrode.

## 2. Materials and Methods

### 2.1. Materials and Apparatus

Na_2_MoO_4_·2H_2_O and L-Cysteine (L-Cys) were purchased from Aladdin Chemical Reagent Company (Shanghai, China). HAuCl_4_ and K_3_[Fe(CN)_6_] were purchased from Sinopharm Chemical Reagent Co., Ltd. (Shanghai, China). Thiourea (NH_2_CSNH_2_) was purchased from Tianjing Damao Chemical Factory (Tianjing, China). Bovine serum albumin (BSA) was purchased from Sigma-Aldrich (St. Louis, MA, USA). The antigen (CH_3_Hg-MNA-BSA) and antibody against Hg (II) were self-made in this experiment. Graphene oxide (GO) was obtained from the Engineering Research Center of Coal-based Ecological Carbon Sequestration Technology of the Ministry of Education of Shanxi Datong University.

Cyclic voltammetry (CV), electrochemical impedance spectra (EIS) and differential pulse voltammetry (DPV) were performed on a CHI660E electrochemical workstation (Electrochemical workstation, CHI660E, Shanghai Chenhua Instrument (Shanghai, China). The experiments were performed on a three-electrode system, with a glassy carbon electrode (GCE) as the working electrode, a platinum wire acting as the auxiliary electrode and a saturated calomel electrode as the reference electrode. X-ray diffraction (XRD, Smart Lab SE, Rigaku, Akishima, Japan) was used to study the crystal structures of the nanomaterials. Characterization of the surface morphology of the nanomaterials was conducted using scanning electron microscopy (SEM, Inspect F50, FEI, Hillsbor, OR, USA). Characterization of the elemental composition of the materials was conducted using X-ray photoelectron spectroscopy (XPS, ESCALAB Xi+, Thermo Fisher, Waltham, MA, USA).

### 2.2. Preparation of MoS_2_, MoS_2_/rGO and MoS_2_/rGO/Au Nanocomposites

MoS_2_ nanoflowers were prepared according to a previous study [30] through a hydrothermal method using Na_2_MoO_4_·2H_2_O and L-Cysteine as precursors. In brief, 0.25 g of Na_2_MoO_4_·2H_2_O was dissolved in 25 mL of ultrapure water, the solution was adjusted to pH 6.5, and after ultrasonication for 5 min, 0.5 g of L-Cysteine and 50 mL of water were added to the solution followed by ultrasonication for 10 min. The mixture was then transferred into a 100 mL Teflon-lined stainless-steel autoclave and reacted at 200 °C for 36 h to obtain MoS_2_ nanomaterials. The product was collected and centrifuged for 30 min at a speed of 10,000 rpm. The resulting black precipitate was washed with ultrapure water and ethanol several times successively and then dried at 60 °C under a vacuum.

MoS_2_/rGO was synthesized as described in the literature [31]. In short, 0.4 g of Na_2_MoO_4_·2H_2_O and 30 mg of GO were dispersed in 30 mL of ultrapure water under ultrasonication. After that, 0.63 g of thiourea was dissolved in the above mixture. The resulting mixture was sonicated for 30 min and then transferred to a 50 mL Teflon-lined stainless-steel autoclave, which was heated to 200 °C for 24 h. The resulting black precipitates were collected by centrifugation and washed with water and ethanol several times and then dried in a vacuum at 60 °C.

MoS_2_/rGO/Au nanocomposites were synthesized via the following method [32]. A total of 10 mg of MoS_2_/rGO nanocomposites was mixed in 10 mL of deionized water, and then 1 mL of 5 mM L-Cysteine was added to be the above solution, which was ultrasonicated for 30 min. After that, 1 mL of a 1 wt% HAuCl_4_ aqueous solution was added into the solution mixture and vigorously stirred for 3 h. After that, the sample was washed with water and ethanol several times, and the precipitates were lyophilized for use.

### 2.3. Preparation of the Modified Electrode

Before preparing the immunosensor, the GCE (3mm in diameter) was first polished with 1 and 0.05 μm of alumina powder and then cleaned thoroughly by sonication before use. Then, 6 μL of a 1 mg/mL MoS_2_/rGO/Au solution was dropped on the surface of the bare GCE electrode and dried, and this was used as the working electrode. For comparison, pure MoS_2_- and MoS_2_/rGO-modified GCE electrodes were prepared using the same procedure as above.

### 2.4. Electrochemical Competitive Immunosensor Measurement

**Scheme 1:**
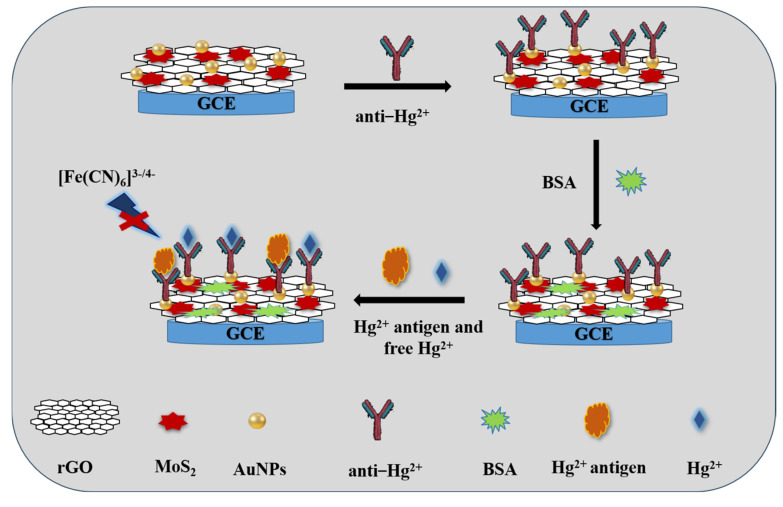
Schematic illustration of the competitive electrochemical immunosensor for Hg (II) based on MoS_2_/rGO/Au nanocomposites.

Scheme 1 shows a diagram of the competitive electrochemical immunosensor. Firstly, 6 μL of Hg (II) antibody dispersion in PBS buffer was dropped onto the modified electrode for 1h. After washing, the electrode was incubated with 1% BSA solution to block the nonspecific binding sites for 30 min. Subsequently, the competitive detection of Hg (II) was performed by incubating the electrode in a 100 μL solution mixture (20 μg/mL antigen + Hg (II) solution with a series of concentrations) for 1h, where the antigen competed for the limited binding sites of the antibody with the Hg (II) in the solution.

The electrochemical immunosensor was measured in 0.01 M PBS (pH 7.4) and 0.1 M KCl containing a 5.0 mM [Fe(CN)_6_]^3−/4−^ solution. The response was calculated with differential pulse voltammetry (DPV) measurement.

### 2.5. Preparation of the Spiked Samples

Lake water samples collected from Wenying lake in Datong city were filtrated using a nylon membrane filter, and the filtrates were adjusted to pH 7.4. Then, the proposed immunosensor was applied to detect the filtrates, which were spiked with Hg (II) at concentrations of 10, 20, 50 and 100 ng/mL.

## 3. Results and Discussion

### 3.1. Characterization of Nanocomposites

The morphologies and structures of the MoS_2_, MoS_2_/rGO and MoS_2_/rGO/Au nanocomposites were characterized by XRD, SEM and XPS.

**Figure 1:**
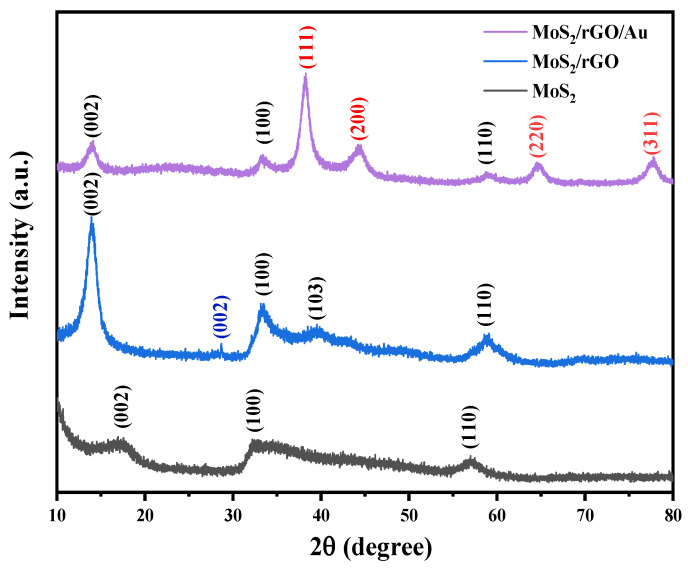
XRD patterns of MoS_2_/rGO/Au, MoS_2_/rGO and MoS_2_ nanocomposites.

Figure 1 shows the crystal structures of the nanocomposites measured by XRD. The peaks at approximately 2θ = 17.1°, 32.8° and 57.4° corresponded to the (002), (100) and (110) planes of the crystalline MoS_2_, respectively (JCPDS 37-1492) [32]. At the same time, there was a peak at 2θ = 28.7°, which was characteristic of the rGO in the MoS_2_/rGO nanocomposite. The relative intensity of rGO was smaller than that of MoS_2_, and a small red shift was observed, which was because of the interaction between the MoS_2_ nanoflowers and the oxygen functional groups on the rGO [28], and the integration of the MoS_2_/rGO was dominated by the MoS_2_ during the synthetic process [33]. The MoS_2_/rGO/Au nanocomposite shows diffraction peaks at 2θ = 38.2°, 44.2°, 64.7° and 77.8°, which corresponded to the (111), (200), (220) and (311) planes of the Au (JCPDS 04-0874). Therefore, the results suggested that the above nanocomposites were successfully synthesized.

**Figure 2:**
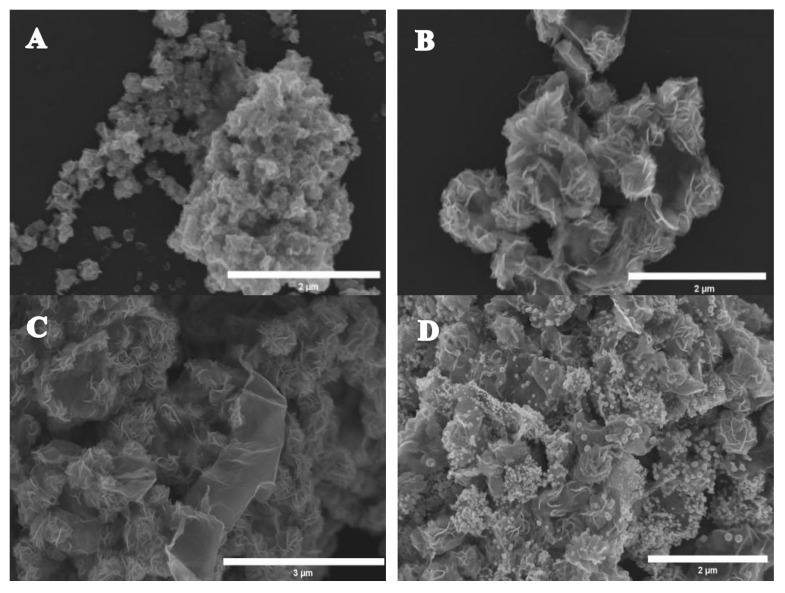
SEM images of (**A**) MoS_2_, (**B**,**C**) MoS_2_/rGO and (**D**) MoS_2_/rGO/Au nanocomposites.

SEM was studied to characterize the morphologies of the MoS_2_, MoS_2_/rGO and MoS_2_/rGO/Au nanocomposites, and the results are shown in Figure 2. As shown in Figure 2A, the thin sheet formed a flower-like nanostructure, indicating the successful synthesis of the MoS_2_. Figure 2B,C show SEM images of the MoS_2_/rGO. It can be observed that large numbers of MoS_2_ nanoflowers were loaded on the rGO’s surface, which was transparent and lamellar and showed a loose porous structure. From Figure 2D, we can see that the small-sized AuNPs obviously decorated the surface of the MoS_2_ and rGO uniformly, which means that the MoS_2_/rGO/Au nanocomposites were successfully synthesized.

**Figure 3:**
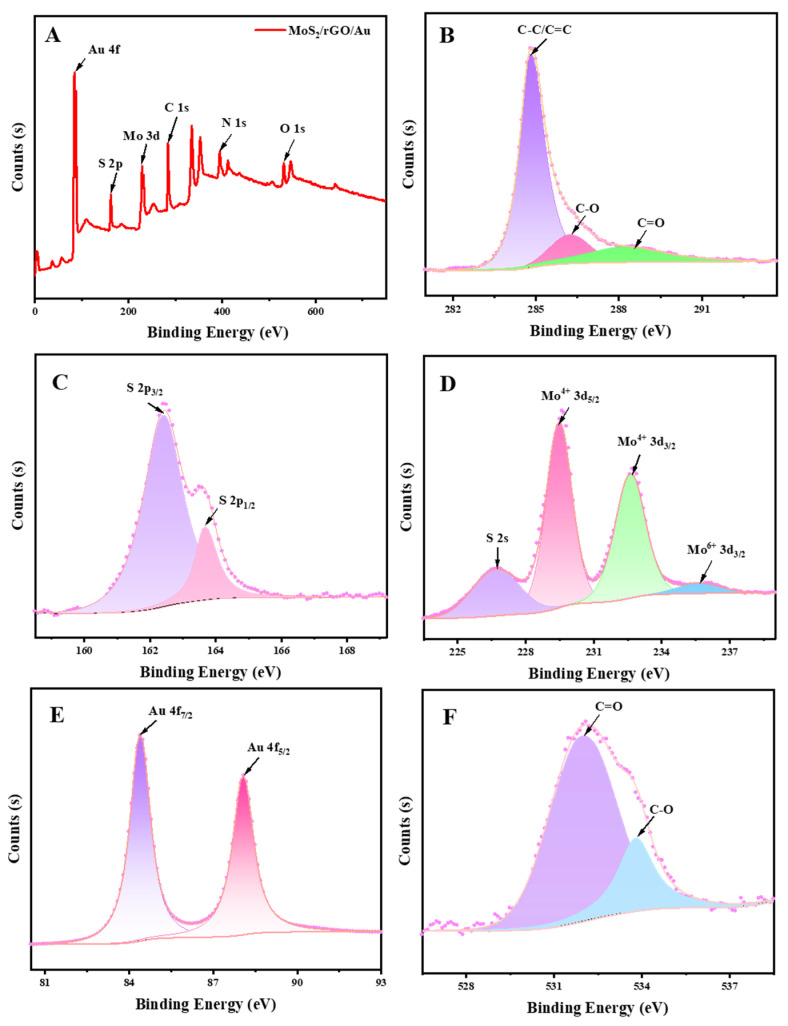
XPS spectra of (**A**) MoS_2_/rGO/Au, (**B**) C 1s region, (**C**) S 2p region, (**D**) Mo 3d region, (**E**) Au 4f region and (**F**) O 1s region.

Figure 3 shows the XPS curves of the MoS_2_/rGO/Au nanocomposites. From the spectrum Figure 3A, we can confirm the presence of Au, S, Mo, C and O in the MoS_2_/rGO/Au. The deconvoluted spectrum of C 1s (Figure 3B) and O 1s (Figure 3F) had peaks located at 284.8 eV, 286.3 eV, 288.6 eV, 531.9 eV and 533.8 eV, corresponding to C-C/C=C, C-O and C=O bonds [34]. The decreased peaks of C-O and C=O indicates the loss of oxygen-containing groups during the reduced procedure of GO. The peaks at 162.4 eV and 163.7 eV in the S 2p region (Figure 3C) imply S 2p_3/2_ and S 2p_1/2_. Figure 3D shows the spectrum for Mo 3d. The peaks at 226.7 eV, 229.5 eV, 232.7 eV and 235.7 eV were related to S 2s, Mo^4+^ 3d_5/2_, Mo^4+^ 3d_3/2_ and Mo^6+^ 3d_3/2_, respectively. The two characteristic peaks at 84.4 eV and 88.1 eV (Figure 3E) corresponded to Au 4f_7/2_ and Au 4f_5/2_, which were loaded on the MoS_2_ and rGO.

### 3.2. Electrochemical Properties of Modified Electrodes

**Figure 4:**
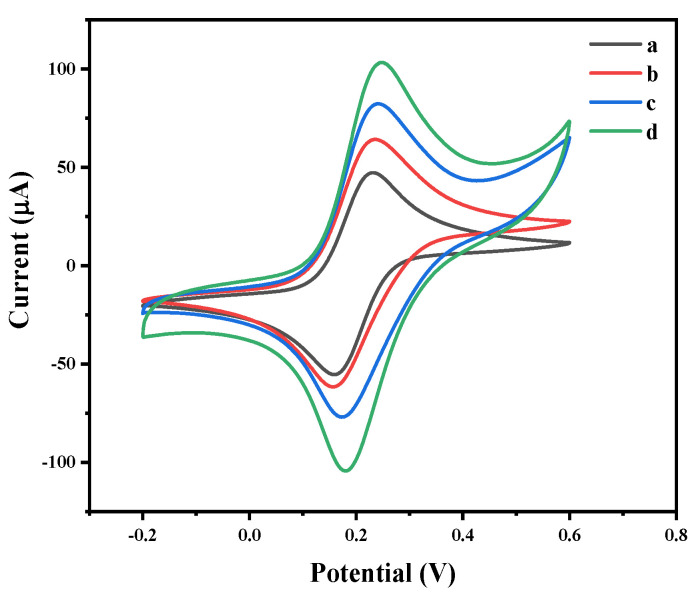
Cyclic voltammograms of (a) bare GCE, (b) MoS_2_/GCE, (c) MoS_2_/rGO/GCE and (d) MoS_2_/rGO/Au/GCE in 0.01 M PBS (pH 7.4) and 0.1 M KCl containing a 5.0 mM ([Fe(CN)_6_]^3−/4−^ solution.

The electrochemical properties of the bare GCE and MoS_2_-, MoS_2_/rGO- and MoS_2_/rGO/Au-modified GCE electrodes were analyzed using cyclic voltammetry (CV). As shown in Figure 4, compared with the bare GCE, the redox peak currents of the MoS_2_-, MoS_2_/rGO- and MoS_2_/rGO/Au-modified electrodes increased gradually, which was attributed to the synergistic electrocatalytic activity of the MoS_2_, rGO and Au [35]. Therefore, the MoS_2_/rGO/Au-modified electrode was used for the following study.

### 3.3. Characterization of Immunosensor

**Figure 5:**
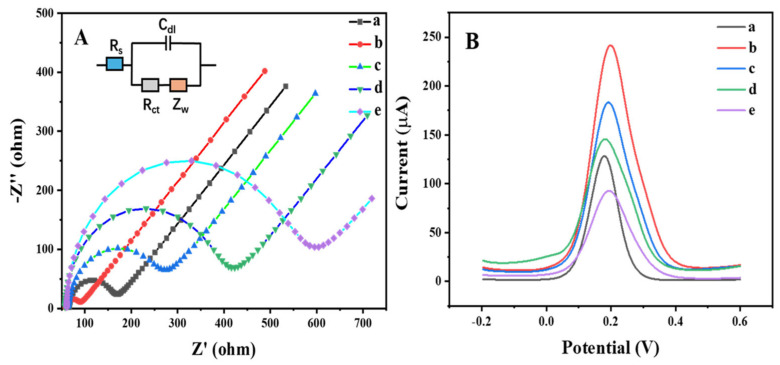
(**A**) Electrochemical impedance spectra and (**B**) differential pulse voltammograms of (a) GCE, (b) MoS_2_/rGO/Au/GCE, (c) anti-Hg^2+^/MoS_2_/rGO/Au/GCE, (d) BSA/anti-Hg^2+^/MoS_2_/rGO/Au/GCE and (e) antigen/BSA/anti-Hg^2+^/MoS_2_/rGO/Au/GCE.

Electrochemical impedance spectroscopy (EIS) is often used to characterize the assembly of each step of the working electrode for immunosensors. As shown in Figure 5A, the impedance spectra included a semicircle at a higher frequency and a line part at a lower frequency; the diameter of the semicircle indicates the electron transfer resistance (Rct) [36]. When the MoS_2_/rGO/Au was coated on the GCE, the semicircle was smaller than the bare GCE, indicating that the MoS_2_/rGO/Au nanocomposites could enhance the electron transfer on the modified GCE. Subsequently, after the Hg (II) antibody, BSA and antigen were immobilized on the modified GCE, the Rct values were increased, respectively, demonstrating that the step-by-step preparation of the immunosensor on the modified electrode was successful.

Meanwhile, the differential pulse voltammetry (DPV) results of the bare GCE and modified GCE were consistent with the EIS results (Figure 5B). The peak current of the MoS_2_/rGO/Au-modified GCE was obviously higher than that of the bare GCE. With the gradual modification by the antibody, BSA and antigen of the MoS_2_/rGO/Au/GCE, the peak current gradually decreased due to the protein layers on the electrode surface significantly hindering the electron transfer.

**Figure 6:**
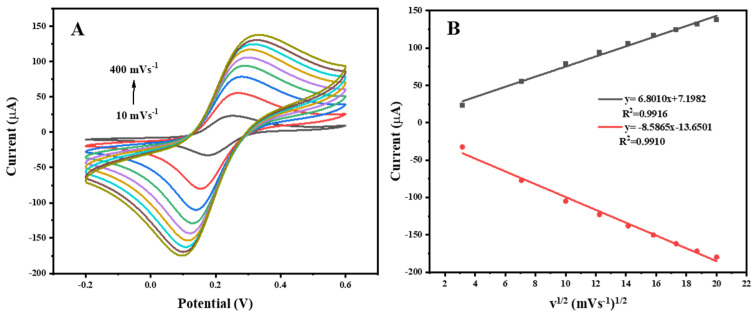
(**A**) Cyclic voltammograms of the BSA/anti-Hg^2+^/MoS_2_/rGO/Au/GCE in 0.01 M PBS (pH 7.4) and 0.1 M KCl containing a 5.0 mM ([Fe(CN)_6_]^3−/4−^ solution at different scan rates ranging from 10 to 400 mV s^−1^. (**B**) The linear relationship between the square root of the scan rate and the peak current.

Moreover, the kinetic process of the electrode reactions was studied. As shown in Figure 6A, the peak current increased with the increasing in the scan rate, and a linear relationship with a good correlation coefficient between the peak currents and the square root of the scan rates was observed (Figure 6B), suggesting that the process was diffusion-controlled.

### 3.4. Optimization of Assay Conditions

**Figure 7:**
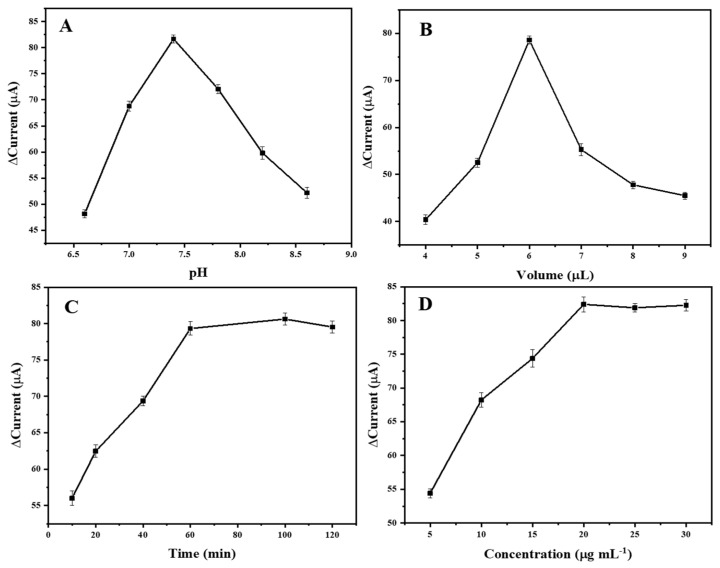
Effects of (**A**) pH, (**B**) amount of MoS_2_/rGO/Au nanocomposite, (**C**) incubation reaction time and (**D**) concentration of antigen.

To establish an optimal electrochemical immunosensor, some parameters, including the pH value, the amount of nanocomposite, the incubation time of the antigen–antibody reaction and the concentration of the antigen, were determined. As shown in Figure 7, the optimal pH = 7.4, the optimal amount of the MoS_2_/rGO/Au nanocomposite was 6 μL (1 mg/mL), the optimal incubation time was 60 min and the optimal concentration of antigen was 20 μg/mL.

### 3.5. Sensitivity of the Immunosensor

**Figure 8:**
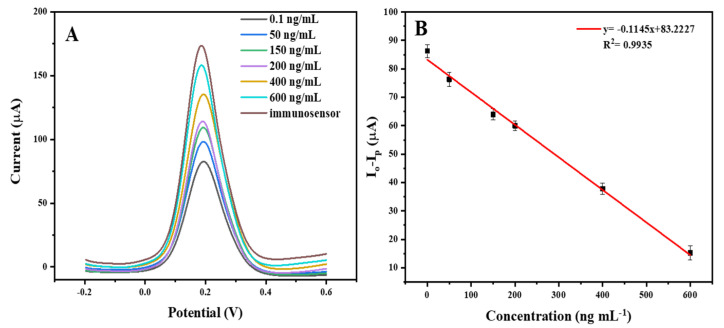
(**A**) Differential pulse voltammetry of the immunosensor before binding and after incubation with different concentrations of Hg (II) mixed with 20 μg/mL of antigen. (**B**) The calibration curve of Hg (II) detection.

For Hg (II) determination, a competitive assay was performed by the incubation of the immunosensor with mixtures of a Hg (II) standard solution (with different concentration) and an antigen solution at the optimal conditions. Figure 8A describes the DPV curves of the immunosensor before and after the immunoreaction. The DPV peak current increased with the increase in Hg (II) in the incubation solution, since the more free Hg (II) in the sample, the less the antigen coul bind to the antibody immobilized on the electrode, and thus the larger DPV current obtained.

The immunosensor response was calculated as the change in the DPV current (i_o_ − i_p_) [37], which was defined as follows: i_o_ is the current before the immunoreaction, and i_p_ is the current after incubating the immunosensor with a mixture of antigen with different concentrations of Hg (II).

Under optimal conditions, the sensor for Hg (II) detection was constructed in the concentration range of 0.1–600 ng/mL. As shown in Figure 8A, the DPV responses increased with the increase in Hg (II). Moreover, there was a good linear relationship between i_o_ − i_p_ and the concentration of Hg (II) (Figure 8B). The limit of detection (LOD) of the immunosensor was 63 pg/mL, which demonstrates the high sensitivity of the proposed method.

### 3.6. Selectivity, Stability and Reproducibility of the Immunosensor

**Figure 9:**
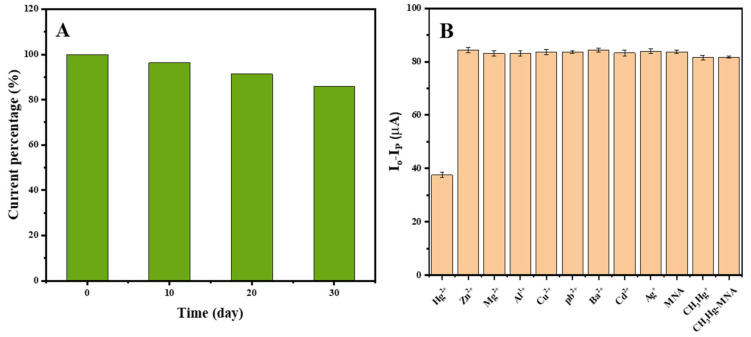
(**A**) The stability and (**B**) selectivity of the competitive electrochemical immunosensor for Hg (II) detection.

The immunosensors were kept at 4 °C over 4 weeks and then tested under optimal conditions. As shown in Figure 9A, compared with the signal before storage, the peak current could still be maintained at 85.76%. The results indicate the good stability of the sensor.

In order to evaluate the specificity of the proposed immunosensor, twelve species, including Zn (II), Mg (II), Al (III), Cu (II), Pb (II), Ba (II), Cd (II), Ag (I), MNA, CH_3_Hg (I) and CH_3_Hg-MNA, were selected as the interference species and applied according to the procedure of the assay. The concentrations of the standard solutions of Hg (II) and interference species were 400 and 1000 ng/mL, respectively. As shown in Figure 9B, the response of Hg (II) was significantly different from the other interference species. This clearly indicates that the electrochemical immunosensor exhibited high specificity in terms of detecting Hg (II).

The reproducibility of the immunosensor was assessed by testing four identical immunosensors for Hg (II) at a concentration of 100 ng/mL, respectively. The relative standard deviation (RSD) was 4.78%, indicating the good reproducibility of the immunosensor.

### 3.7. Spiked Samples Analysis

Recovery experiments in lake water samples were carried out to evaluate the reliability of the sensor. Table 1 shows the results of the recovery and RSD. It was observed that the recovery of the spiked samples ranged from 98.4% to 100.3%, and low RSD values were obtained. These results indicate that the proposed method could be used for the detection of real samples.

## 4. Conclusions

In summary, a sensitive and specific MoS_2_/rGO/Au-based competitive electrochemical immunosensor for the detection of Hg (II) was developed successfully. The MoS_2_/rGO/Au nanocomposites displayed excellent electrochemical properties due to their synergistic effects, and the antibody could be adsorbed by the Au in the nanocomposites. The sensor has not only good selectivity, stability and reproducibility for Hg (II) but also has a low LOD value of 63 pg/mL. The proposed method was used for the detection of Hg (II) in spiked water samples with an acceptable recovery rate. Therefore, this approach provides a sensitive and specific analytical method for the detection of Hg (II), and it has good prospects as a useful model for the detection of other small-molecular compounds in the food safety and environmental areas. Even so, it is relatively important to screen the immune reaction conditions of the electrode, such as the pH, the amount of nanocomposite, the incubation time and other conditions. Otherwise, they will affect the occurrence of the incubation reaction, which is also the focus of our future work.

## Figures and Tables

**Table 1:** Results of recovery and relative standard deviation (RSD) for Hg (II) determination from fortified samples.

Fortified (ng/mL)	Measured (ng/mL)	Recovery (%)	RSD (%) (*n* = 3)
10	9.88 ± 0.34	98.8	3.44
20	19.89 ± 0.51	99.5	2.56
50	49.22 ± 2.64	98.4	5.36
100	100.34 ± 4.59	100.3	4.57

## Data Availability

Data are contained within the article.
